# Fibromyalgia Syndrome: A Metabolic Approach Grounded in Biochemistry for the Remission of Symptoms

**DOI:** 10.3389/fmed.2017.00198

**Published:** 2017-11-13

**Authors:** Silvia Maria Lattanzio

**Affiliations:** ^1^Department of Biomedical Sciences, University of Padova, Padova, Italy

**Keywords:** fibromyalgia syndrome, tryptophan, serotonin, fructose malabsorption, fibromyalgia symptom remission, chronic pain, metabolism

## Abstract

Fibromyalgia syndrome (FMS) is a chronic, complex, and heterogeneous disorder of still poorly understood etiopathophysiology associated with important musculoskeletal widespread pain, fatigue, non-restorative sleep, and mood disturbances. It is estimated to afflict 2–3% of the worldwide population, with clean prevalence among women. The objective of this paper is to propose a novel treatment for symptomatic remission of FMS, grounded in biochemistry and consisting in the withdrawal from the diet of molecules that can indirectly trigger the symptoms. The hypothesis develops from the evidence that low serotonin levels are involved in FMS. Serotonin is synthesized starting from the essential amino acid tryptophan. The presence of non-absorbed molecules in the gut, primarily fructose, reduces tryptophan absorption. Low tryptophan absorption leads to low serotonin synthesis that triggers FMS symptoms. Moreover not-absorbed sugars could also produce a microbiota deterioration activating a positive feedback loop: the increasing microbiota deterioration reduces the functionality of absorption both of fructose and tryptophan in the gut, entering a vicious circle. The therapeutic idea is to sustain serotonin synthesis allowing the proper tryptophan absorption. The core of the cure treatment is the exclusion from the diet of some carbohydrates and the marked reduction of some others. The main target is the limitation of total dietary fructose as marked as possible. It could be an effective strategy to get the remission of symptoms acting on the impaired biochemical pathways. The straying from the treatment is expected to cause the reappear of the symptoms.

## 1 Introduction

Fibromyalgia Syndrome (FMS) is a chronic, complex, and heterogeneous disorder (1, 2) of still poorly understood pathophysiology (1, 3, 4) associated with important musculoskeletal widespread pain, fatigue, and mood disturbances (1, 2). It is estimated to afflict 2–3% of the worldwide population, with clean prevalence among women (4). The constellation of symptoms that characterize this condition comprises lower pain threshold to normally non-painful stimuli (allodynia), greater sensitivity to pain stimuli (hyperalgesia), stiffness, fatigue, psychological distress (depression), cognitive impairment, such as problems with short-term memory, impaired speed of information processing, limited multi-tasking performance, and reduced attention span (1–3). Moreover, the majority of patients has one or more pain co-morbidities or associated disorders: among them lower back pain, specific regions of localized tenderness, non-restorative sleep and sleep disturbances, irritable bowel syndrome (IBS), restless leg syndrome and leg cramps, headache, migraine, visceral pain, temporomandibular disorder (TMD), anxiety, palpitation, chest pain and muscle twitching (2–7).

FMS still has no effective cure (1). Pharmacological and non-pharmacological therapies to control symptoms have limited effectiveness (1). The recent “revised recommendations for the management of fibromyalgia” concluded that “the most of them are ‘expert opinion’ and are not supported by strong evidence from the scientific literature.” The only ‘strong for’ recommendation is exercise (1). Often co-morbidities and associated disorders are managed with additional treatments (8), demonstrating the lack of understanding of the real source of the abnormal nervous system sensitization. “FMS has substantial impacts on the physical functioning, the mental health, and the quality of life of those suffering from it” (9) as well as their relatives, “together with direct economic costs” (9, 10), (as medical care expenses and non-medical associated costs), and “indirect costs in term of productivity losses” (9). FMS diagnosis and management thus is still a substantial challenge for patients, physicians (1), and society as a whole for its associated economic burden (9).

The objective of this paper is to propose a novel treatment for the remission of symptoms of FMS, clearing up the logical steps that support the idea addressing its foundations grounded in biochemistry. An hypothesis of the primary cause of FMS is also proposed.

## 2 Rational of the Treatment Hypothesis

The apparent heterogeneity of the symptoms is the key information for catching the core of FMS. It has to be considered a disease as a whole. It is correctly defined, indeed, a syndrome. Looking at the common cause that connects all the symptoms, instead of searching for a managing strategy of each one apart, is the effective way to understand the impaired biological pathways and to built a remission cure.

The common denominator among all the symptoms is serotonin (5-hydroxytryptamine, 5-HT). This is the starting point of the deductive reasoning that led to investigate biochemical and physiological mechanisms that are the cure foundations. In short, the logical steps that constitute the rational of the idea are the following:

- low 5-HT levels are involved in FMS (11, 12);
- 5-HT is synthesized starting from the essential amino acid l-tryptophan (Trp) (13);
- the presence of non-absorbed molecules in the gut, primarily fructose, reduces Trp absorption (14);
- low Trp absorption reduces Trp bioavailability;
- low Trp levels reduces 5-HT biosynthesis;
- low 5-HT levels trigger FMS symptoms.

The presence of non-absorbed fructose could also produce a microbiota deterioration activating a positive feedback loop: the increasing microbiota deterioration reduces the functionality of the absorption both of fructose and Trp in the gut, entering a vicious circle. Moreover, it could affect the absorption of other essential amino acid, as Maillard reaction is not restricted to Trp, and the absorption of some mineral salts (14). It could not be excluded that other molecules, like sorbitol or other sugars, that do not have specific transport system may produce similar effects.

## 3 Biochemical Background

### 3.1 Tryptophan

Trp is an essential amino acid for humans: its availability as a substrate for biosynthesis relies on dietary intake (13). Moreover, its bioavailability is influenced by several factors such as the presence of other essential amino acids (15). Trp is involved in crucial metabolic pathways that results in various end-products, among them proteins, the neurotransmitter 5-HT and kynurenines such as quinolinic acid, 3-hydroxykynurenine and kynurenic acid. Two are the non-protein viae of Trp metabolism: kinurenine (KYN) (16, 17) and methoxyindole. The most of Trp (at least 95%) is metabolized via the KYN pathway. The reaction is catalyzed by the rate-limiting enzymes Trp 2,3-dioxygenase (TDO) or indoleamine 2,3-dioxygenase 1 (IDO1) and generates immunomodulatory and neuroactive metabolites. The methoxyindole pathway is the one where 5-HT synthesis occurs (13).

“Once absorbed by the body, Trp travels around the periphery circulation, either bound to albumin or in free form. The two states are in equilibrium, with the former accounting for up to 90%” (16). However, “Trp can only be transported across the blood–brain barrier in its free form by the competitive and non-specific l-type amino acid transporter” (16). “Trp reaches the brain when ingested food is rich in this amino acid and not excessively rich in other large neutral amino acid (LNAAs), that could compete with it” (15).

### 3.2 Serotonin

5% of the available Trp is used as substrate to synthesize the biogenic monoamine 5-HT in a two-step reaction via the methoxyindole pathway (17). The first step is catalyzed by the rate-limiting enzyme tryptophan hydroxylase (TPH), existing in human body in two isoform (TPH1 and TPH2) (18) and leads to the intermediate products 5-hydroxy-tryptophan (5-HTP) (13). The second step is controlled by 5-hydroxytryptophan decarboxilase and leads to 5-HT. The final step of the methoxyindole pathways is controlled by the enzymes monoamine oxidase (MAO) and aldehyde dehydrogenase that lead to 5-hydroxyindole acetic acid (5-HIAA) finally excreted in the urine (13).

Despite its well-known role in the CNS, only approximately 5% of body 5-HT is found in the brain, thus it is important to remember its production and actions in the periphery (19). “Enterochromaffin (EC) cells produce and secrete far more 5-HT than either central or peripheral serotonergic neurons to reach the GI lumen and blood” (20). Indeed, 95% of body 5-HT is found in the gastrointestinal (GI) tract: 90% in EC and 10% in serotonergic neurons of myenteric plexus (20). GI tract is the sole source of blood 5-HT, taken up by 5-HT transporter (SERT) (19).

The serotonergic system is involved in the regulation of many neurophysiological processes and behavioral functions, among them nociception, sensory function, appetite, gastrointestinal function, motor function, mood, cognition, sleep, sexuality, neuroendocrine function (19). 5-HT also plays diverse roles in cardiovascular system (21) and its contribution is emerging in communication between CNS and immune system (19).

### 3.3 Fructose

d-Fructose (fructose) is a hexose sugar naturally present in fruit and honey. In the last decades its consumption is making an increasing contribution to the Western diet since it is used in industrial products as sweetener in the form of high fructose corn syrup (22). The transport of fructose into and out cells in human body is mainly mediated by three proteins: GLUT5, GLUT2, and GLUT7 transporters, characterized by different substrate specificity and tissue expression. Minor contribution to fructose transport is reported also for the other transport proteins in Class II, that exhibit sequence similarity to GLUT5 and 7: GLUT9 and GLUT11 (23). GLUT5 is a low-capacity transporter, glucose independent and specific for fructose. It is mainly expressed in the jejunal region of the small intestine, but also in kidney and brain. GLUT2 is glucose-dependent low-affinity fructose co-transporter. GLUT7 transports glucose and fructose with high affinity for both (23). GLUT7 has the closest sequence similarity to GLUT5 and is primarily expressed in the distal region of the small intestine, ileum, and colon (24).

Different factors may influence the absorption of free fructose: alterations in the functional capability of GLUT5 may influence the absorption efficiency and a role might also be played by dietary intake of fructose and sucrose too, that influence the transporter expression (25). Moreover the expression of the GLUT5 gene seems to have a diurnal variation (26). Furthermore, the co-ingestion of glucose or galactose considerably enhances fructose absorption activating apical GLUT2 mechanism (25).

Fructose is present in human diet in three forms: as pure monosaccharide, as disaccharide sucrose, in complex with glucose, and in the polymerized forms as oligosaccharides and polysaccharides (25). Chains of fructose units with a terminal glucose molecule are called fructans (27), but some authors reserve the term only for longer fructose chains classified into the class of polysaccharides (28). Thus, fructose chains with a 2- to 9-unit length are variably referred to as oligofructose (27) or fructo-oligosaccharides (FOS) (28) and those with ≥10 units as inulins or fructans (27, 28). Human ability to break down oligo- or polysaccharides in the small bowel is limited and only 5–15% of fructan can be absorbed (27). It is due to the lack of enzymes to fully hydrolyze glycosidic linkages in the complex polysaccharides, determining the presence of non-absorbed fructans in the colon (27).

## 4 Evaluation of the Hypothesis

### 4.1 The Crucial Role of Diet

Interesting results confirm the crucial role of food intake on 5-HT availability and the biochemical and physiological mechanisms that directly or indirectly determine FMS symptomatology.

#### 4.1.1 Fructose Malabsorption

“Fructose malabsorption is characterized by the inability to absorb this molecule efficiently. As a consequence fructose reaches the colon where it is broken down by bacteria to short fatty acids, carbon dioxide, hydrogen, methane, and lactic acid. Bloating, cramps, osmotic diarrhea and other symptoms of irritable bowel syndrome (IBS) are the consequence” (29). Interestingly a significant percentage of patients with fructose malabsorption are asymptomatic (29), even during the fructose challenge (22). It means that gastrointestinal symptom profiles *per se* may be insufficient to diagnose fructose malabsorption. On the other hand, “symptoms as abdominal pain, bloating, flatus and discomfort producing by excessive amount of gases such as hydrogen, carbon dioxide, methane and short-chain fatty acids, are not specific for fructose intolerance but are similar to the symptoms observed in patients with sorbitol or lactose intolerance” (22). The adverse effect of dietary fructose that a subject may experience depends both on its amount and personal tolerance. It is important to stress that the efficiency of fructose absorption can be much variable among subjects and influenced by several condition: among them, the amount of total ingested fructose, in fact, GLUT5 is a low and saturable transporter (30); the concurrent presence of glucose, which activates the GLUT2 co-transporter (28); the expression of the transporter.

Most dietary fructose is completely absorbed in normal subjects, when ingested in small quantities (31). However, in modern Western diets fructose consumption can reach levels at which malabsorption occurs even in the healthiest subjects (25, 31). When fructose is present in excess of glucose (referred to as “free fructose”) and the capacity of the GLUT5 transporter is lower or impaired, fructose is malabsorbed, a physiological phenomenon that is estimated to be present at least in one-third of adult population (28). The causes responsible for fructose malabsorption are unknown and still a matter of debate (20, 32–35). GLUT5 gene mutations on exons (33) or reduced GLUT5 gene expression (32) were hypothesized as responsible for gastrointestinal symptoms, but these hypotheses were not confirmed by the experimental results obtained in the few studies performed on patients with functional gastrointestinal disorders (32) and children with fructose malabsorption (33). Attention could be focused not only on the transporter expression in the cytosol, but also on to the translocation capacity of the protein. An impairment in the process of insertion of GLUT5 into the brush-border of the apical membrane of enterocytes could be hypothesized.

The negative implication of fructose malabsorption may also be responsible for impairment in mineral salt absorption. An interesting study reports association between low serum zinc concentrations and fructose malabsorbers (36). Gastrointestinal track maybe one of the target areas where zinc insufficiency has relevant consequences (37). The negative impact on plasma iron and ferritin, instead, showed not significant differences; nevertheless, a tendency toward lower concentrations in fructose malabsorbers was reported (36).

Symptoms of fructose malabsorption may be exacerbated by the sugar alcohol sorbitol (28): in fact, it undergoes only slight intestinal resorption by passive diffusion and it is also able to directly inhibit GLUT5 transporter (30).

#### 4.1.2 Tryptophan Depletion

The method of acute tryptophan depletion (ATD) reduces Trp availability as substrate for 5-HT synthesis (15). Positron emission tomography (PET) study under ATD condition demonstrate “a marked lowering of brain 5-HT synthesis in all brain regions examined” and the effect in the brain seems to be greater than in free plasma Trp (38). Variations in food intake of Trp can have profound effects upon the 5-HT synthesis and “may impact upon those aspects of brain function that are influenced by serotonergic neurons” (15). It was reported that “diets low in Trp produce Trp deficiency in only 2 days” and “women appear to be more susceptible to these changes which may be explained by differences in the metabolic pathways of Trp in females” (39). The hypothesis proposed by the authors is linked to the enzymatic pathway that “leads to the synthesis of KYN instead of 5-HT formation” related to the “greater activity of the liver enzyme Trp-2,3-dioxygenase, which is estrogen dependent” (39). In fact, “no differences have been found between the number of 5-HT reuptake sites in the brains of normal male and female subjects … moreover males and females seem to have similar stores of brain 5-HT, but the mean rate of 5-HT synthesis in normal males were found to be 52% higher than in normal females” (38). If an increased utilization of 5-HT is required, “a lower rate of synthesis in females may not be as efficient in maintaining adequate stores of the neurotransmitter” (38). Thus, during stressful situations, “5-HT levels would decline more in female than in male subjects, possibly increasing vulnerability to depression” (38).

It was reported that the depletion of 5-HT precursor Trp by dietary exclusion of this amino acid has caused clinical deterioration of patients with major depressive syndrome (39). Ledochowski et al. describe the association between lactose malabsorption and depression in female patients suggesting that “lactose malabsorption may play a role in the development of mental depression” (40). It was proposed by the authors that high intestinal lactose concentrations in the gut of lactose malabsorbers might interfere with Trp metabolism, reducing 5-HT availability as consequence (40). The physio-pathological mechanismproposed is the formation of complexes between non-absorbed lactose and Trp that has a negative effect on the absorption of Trp (39).

Similar results were obtained in patients with fructose intolerance (41) and the concomitant role of sorbitol was addressed too (42). Lower plasma Trp concentrations were observed in subject with fructose malabsorption and higher scores in depression inventory evaluations compared with subjects with normal fructose absorption (29). Moreover, under stress condition, release of cortisol could upregulate activity of Trp to KYN metabolism reducing the substrate for 5-HT synthesis (39).

#### 4.1.3 Maillard Reaction

The formation of a fructose–Trp complex (14) was previously proposed as an explanation for the possible association between fructose malabsorption and disturbed Trp metabolism (29). It was proposed that fructose malabsorbers have greater probability for forming the non-absorbable fructose-Trp complexes due to the presence of high fructose concentrations in the gut (41). “As a consequence not only fructose but also Trp is absorbed to a lesser extent” (41).

The chemical basis of these hypotheses are the well-known Maillard reactions that occurs between sugars and amino groups. Reducing sugar, fructose in primis, is involved in these non-enzymatic reactions with proteins: decreased protein quality due to the loss of amino acid residues and decreased protein digestibility are the consequences (14). Maillard reactions can also have negative effects on the metabolism and absorption of other nutrients, including minerals, such as zinc (14). Particularly, the effect on zinc level could be crucial, as it seems to be involved in cellular turnover of the gastrointestinal mucosa (36).

The concentration of the reducing sugar and its degree of reactivity determine the rate at which the Maillard reactions occur (31). This way the high reactivity of fructose due to the fact that it exist to a greater extent in the open-chain form (14) would explain its major contribution in glycosilation reaction, even in case of the circulating concentration of fructose is substantially lower than that of glucose (31). *In vitro* data demonstrate that the rate of protein cross-linking in the presence of fructose is 10 times greater than in the presence of glucose (31). Thus, even though absolute fructose concentrations remain low in comparison with glucose concentration, it was not excluded that the large percentage increases in serum fructose or sucrose might have clinical consequences (31). Finally, fructose promotes the formation of advanced glycation end-products (AGEs) to a considerably greater extent than other reducing sugars (e.g., glucose and lactose), so that a marked reduction in its consumption could have beneficial health effects at all (31).

## 5 Discussion

The distance among symptoms of FMS is only apparent and the common denominator among them is clearly 5-HT. This neurotransmitter plays a leading role in human body, regulating critical functions: besides pain sensitivity and sensory function, mood and cognition, sex and sleep, even appetite, emesis, endocrine function, gastrointestinal function, motor function, neurotrophism, and vascular function are under its control (19). So, it is clear that an impairment in 5-HT pathway has a great potential for triggering a disease condition involving different apparatuses. Since 5-HT is synthesized from the essential amino acid Trp, an impairment in Trp absorption is expected to have an impact on 5-HT synthesis.

### 5.1 Understanding FMS Pathophysiology

The core of the hypothesis is that a gastrointestinal malabsorption has a central role as a primarily cause of FMS and that it is mainly due to fructose. This way, the core of the treatment is the contrast of Trp malabsorption, favoring its normal absorption.

At the very beginning, the hypothesis springs from the observation of marked worsening of pain and stiffness in a FMS patient after a breakfast of fruit, marmalade, and rye bread leading to the hypothesis of the involvement of fructose as symptom trigger. The data of decreased serum Trp concentrations in fructose malabsorbers supports the view that fructose malabsorption interferes with Trp metabolism (29). Moreover, the concept that an impairment of Trp absorption can be related to sugar malabsorption is supported by evidences that link lactose and fructose malabsorption to depression, reported in few but significant observations in the literature (29, 39–41).

The implication of the presence of fructose may be not limited to Trp malabsorption but could affect the absorption of other essential amino acids, since Maillard reactions occur not only in the presence of Trp: some experimental data converge in this direction. Maes et al. reported a significantly lowered plasma concentrations of valine, leucine, isoleucine, and phenylalanine in FMS patients than in controls (43) and similar results on an altered amino acid homeostasis were reported by Bazzichi et al. (44). Furthermore, the negative implications of Maillard reactions may have a crucial role on the absorption of some minerals, such as zinc (14). The involvement of zinc in many biochemical pathways (45) reveals its importance for human health and the hidden consequences that fructose malabsorption could directly and indirectly have. In fact, “a prolonged low zinc intake deprives the organism of the local potential beneficial effects of zinc, including interactions with oxidative free radicals and nitric oxide metabolism” (37).

So, the presence of non-absorbed molecules in the gut could also directly or indirectly produce a microbiota deterioration activating a positive feedback loop: the increasing microbiota deterioration reduces the functionality of absorption both of fructose and Trp in the gut, entering a vicious circle. Apart from the order of the events the result is the same: the reduction of Trp absorption and 5-HT synthesis as consequence.

My main hypothesis to explain the fructose malabsorption condition is a reduced transport capacity of GLUT5 due to a mutation able to impair the functionality of binding. Indeed, a single point mutation in the substrate-binding site would be enough to switch the substrate-binding preference of GLUT5 from fructose to glucose (46).

Although fructose malabsorption plays a primarily role, it appears reasonable that it is not the sole cause of FMS, mostly because the incidence of fructose malabsorption (28) is well greater than the FMS ones (4). Maybe, it can just be considered the main risk factor for FMS.

### 5.2 Treatment Guidelines

The therapeutic idea for the symptomatic remission of FMS is to sustain serotonin synthesis allowing proper Trp absorption, or reversely: to allow Trp absorption to guarantee its bioavailability for 5-HT synthesis. The core of the cure treatment is the exclusion from the diet of some carbohydrates and the marked reduction of some others. Given that fructose is a high reactive sugar, it is the major target of the treatment. The essential is the limitation of total dietary fructose as marked as possible, not neglecting the role of other molecules, such as sorbitol and also fructose chains, as fructans and inulins. The essence of the treatment is removal of what is bad but ensure what is good. Theoretically, the most efficacious management would be a fructose free, fructan restricted, lactose free, sorbitol free, aspartame free, low sucrose diet, together with proper Trp intake.

Given its foundations, the cure is expected to obtain a remission, not a final recovery. The straying from the treatment is expected to cause the reappearance of the symptoms.

Periodic blood exams should be considered to assess vitamin and mineral salt levels before, during, and after the clinical implementation of this protocol. Proper vitamin supplements and mineral salt supplements should be taken into consideration if deficiency are revealed.

### 5.3 The Diet Management

As already mentioned, the target is minimizing the presence of not-absorbed molecules in the gut, removing them from the diet, up to the complete withdrawal in severe FMS conditions.

Due to the fact that fructose is a high reactive reducing sugar (14), it is the major responsible for the impairment of Trp absorption in the gut. Anyway, the role of other sugars has to be considered and will be briefly addressed.

The presence of not-absorbed fructose may have different causes: the easiest one to face and solve is the fructose overload. Reducing its intake is the fastest and easiest way to reduce the symptoms; the second cause is fructose malabsorption, maybe due to a condition of failure in the transporter protein conformation or acquired weakness that impairs the substrate-binding, leading to a low capacity of the transporter. Avoid free fructose intake, reduce fructans intake, and balance the amount of fructose, eventually ingested, with the co-ingestion of glucose to activate the GLUT2 co-transport via, is the way to face up this condition. Excessive amount of fructose is very easy to be reached, as modern Western diets are rich in fructose due to industrial food containing high fructose corn syrup (HFCS), fresh fruit that naturally contains fructose, honey, soft drink, and beverages containing HFCS and saccharose (27, 28, 34, 47). In addition, wheat and the most of cereals, the most of legumes and many vegetables contain fructans (28), contributing to increase the total amount of ingested fructose.

Lactose might have the same consequences of fructose in the gut, especially if the enzyme lactase is deficient.

Disaccharide sucrose would be a separate matter, because in this case fructose is naturally present in equimolar concentration to glucose. This way the pathway of the high-capacity glucose-dependent fructose co-transporter GLUT2 is activated, considerably enhancing fructose absorption (25). The contribution of sucrose malabsorption *per se* to Trp malabsorption and/or microbiota deterioration can be supposed less relevant, in fact the deficiency of the enzyme sucrase is a very rare congenital condition except in Greenland (25).

The other hexose sugars, xylose and arabinose, are passively absorbed (28), this way they might be potentially involved in Maillard reactions, and their withdrawal is suggested.

Despite structural similarities with fructose, sorbitol lacks a specific transport system and it has only passive absorption (28). Its withdrawal from the diet is suggested too, since even glucose co-ingestion does not facilitate sorbitol absorption. Sorbitol exacerbates symptoms in fructose malabsorbers (42), as it directly inhibits GLUT5 (30). Artificial sweeteners and confectionery food often contain sorbitol and the other polyols (27).

Trp supplements (like Trp tablets) are not suggested in any way, as their intake has potential for important side effects by themselves or due to the interaction with serotonergic drugs, up to life-threatening effects related to serotonin syndrome (48).

Suggested food containing Trp are: meat, eggs, fish, rice, potatoes, dark chocolate, and walnuts.

A further note concerns monosodium glutamate (MSG) and aspartame: their withdrawal from the diet is suggested too. MSG naturally exerts excitatory action in both the central (49) and peripheral nervous system (50). Nevertheless, when artificially introduced in food, it can have excitotoxic effects (51). Aspartame might significantly impair the release of 5-HT in the brain (52). “It can increase the supply of phenylalanine, which subsequently can promote a decrease in Trp uptake by brain tissue or a depression in Trp conversion to 5-HT” (52).

The evaluation of this restrictive diet as possibly inappropriate from a nutritional perspective must be properly considered. The withdrawal of fructose-containing food could lead to a concomitant reduction of vitamin and mineral salt intake. Periodic blood exams must be considered to evaluate their levels: vitamin and mineral salt supplements must be considered to address deficiencies already present and to prevent possible ones during and after the protocol implementation.

### 5.4 On the Prevalence of FMS among Women

The Marked difference among sexes in the rate of 5-HT synthesis (38) may be the most relevant factor to explain the lower incidence of FMS in males, once more supporting the crucial role of 5-HT synthesis in FMS pathophysiology. In fact, males and females seem to have similar stores of brain 5-HT, but the mean rate of 5-HT synthesis is significantly lower in women.

Nevertheless, some other considerations can be made. The association between decreased serum Trp concentrations and sugar (fructose and lactose) malabsorption was observed in experimental studies significant only in women. This could be a consequence of the greater activity of the hepatic enzyme TDO, which is estrogen dependent (39, 40). In fact, estrogens have a stimulatory effect on the liver enzyme TDO, thus shifting the Trp metabolism away from the 5-HT pathway and toward the KYN ones (40).

Stress may also provoke similar effect activating the same biochemical pathway, contributing in the exacerbation of patient conditions: besides estrogens, indeed, TDO is inducible by Trp itself and glucocorticoids (53). The glucocorticoid cortisol is rapidly release in response to emotional stress, stimulating the Trp-KYN pathway activity (52), once more reducing the availability of Trp for 5-HT synthesis.

Last but not least, since the expression of GLUT5 occurs primarily in the small intestine, but lower levels are also expressed in the cells of testes, skeletal muscle (24), and adipose tissue (23), an impairment or reduced capacity of this transporter will have a major impact on women, since skeletal muscle amount is on average less than in men and testes obviously lack.

## 6 Conclusion

This paper presents the hypothesis grounded in biochemistry of a novel treatment for the remission of FMS symptoms related to the hypothesis that a gastrointestinal malabsorption has a central role as a primarily cause of FMS and that it is mainly due to fructose.

The novel remission treatment is a strict dietary strategy. It is low cost and drug free, so intrinsically not prone to collateral effects related to drug intake (54). The core of the treatment is the withdrawal from the diet of molecules that might indirectly trigger the symptoms reducing Trp absorption and, as consequence, its availability as substrate for 5-HT synthesis. The diet in practice turns into the avoidance of foods containing substantial free fructose and the restriction of intake of fructans: the main target is the limitation of total dietary fructose as marked as possible. Theoretically, the most efficacious management would be a fructose-free, fructan-restricted, lactose-free, sorbitol-free, aspartame-free, and MSG-free diet together with proper Trp intake, in severe cases a near to zero fructans intake would be necessary, at least for a period.

Given its foundations, the cure is expected to obtain a remission not a final recovery. The straying from the treatment is expected to cause the reappearance of the symptoms.

This paper suggests only Trp intake through diet, maximizing its absorption potential, avoiding as much as possible its co-ingestion with fructose-containing food, in order to guarantee its availability for 5-HT synthesis, maximizing the positive effects of its nutritional properties. The role of some other sugars should be taken into account, as they could determine the same consequences of fructose on Trp absorption.

The remission is a short-term result and is related to be or not on diet guidelines: small indiscretions might have great potential for the return of symptoms in severe conditions. The effect on reducing microbiota deterioration, instead, could be a long-term result, but experimental data from long-term studies will be necessary.

Finally, the crucial role of 5-HT in the body, 5-HT interaction with immune system, the possible interference of fructose with the absorption of essential amino acid and other nutrients and the possible role of non-absorbed fructose in the microbiota deterioration suggest that fructose malabsorption may have really more relevant effects on human health then considered up to now.

## Author Contributions

The author confirms being the sole contributor of this work: developed the hypothesis, researched, wrote the paper, and approved it for publication.

## Conflict of Interest Statement

The author declares that the research was conducted in the absence of any commercial or financial relationships that could be construed as a potential conflict of interest.
